# No one is talking about the elephant in the room

**Published:** 2016-10-18

**Authors:** Marcel D’Eon

**Affiliations:** University of Saskatchewan, Saskatoon, SK

This issue of the CMEJ contains several very interesting studies exploring a range of topics and groups. Two articles touch on culture - patterns of beliefs and practices held in common that provide us with shortcuts in making decisions but which at the same time constrain (and maybe warp) us. The commentaries on medical student entitlement are short but powerful pieces well worth the time to wrestle with them. Two articles address life after residency: clinician educators’ work-work balance and physician recruitment. The bulk of the articles contained in this issue centre on minor curricular innovations and improvements. I want to expand on what Danilewitz and colleagues found when asking about incorporating leadership training into residency education: There’s no time! This is the elephant in the room; the one that no one wants to address or confront. It takes up space and makes us all behave in ways so as not to rile the beast. Let me translate to make this perfectly clear: “there’s no time” really means there are other topics and activities of higher priority. “They are busy!” means, “They have more important things to do, sorry!” So what is more important than leadership training, point of care ultrasound, transgender health, exercise prescription, and more emphasis on decision-making? Most everything we already do, it seems; medical education is an organism without an excretory system.

Marcus Law and colleagues start us off in the dark and murky world where social media and professional expectations mingle with “Exploring Social Media and Admissions Decision-Making – Friends or Foes?” Their study explored social media in Canadian undergraduate admissions, and the attitudes of admissions personnel towards such use. A sizeable proportion of admissions personnel had examined social media profiles at some point to acquire information on applicants. There seems to be an ethical vacuum, and participants expressed significant apprehension based on concerns for fairness and validity. What happens in admissions stays in admissions?

“Attitudes of Canadian Psychiatry Residents If Mentally Ill: Awareness, Barriers to Disclosure, and Help-seeking Preferences” by Tariq Hassan et al. show that the medical culture defined by mental illness stigma, non-disclosure, and avoidance of professional treatment - like a good zombie - just won’t die! One third of their respondents (psychiatry residents at all levels) admitted to a personal history of mental illness. Frequent barriers to disclosure included stigma, career implications (more stigma?), and professional standing (which sounds like stigma yet again). How many orthopedic residents would tell a friend they had a broken leg before going to see a surgeon for fear their career might be negatively affected by their rather obvious, unsightly, and debilitating cast?

Liao, a medical student when he wrote “Entitlement and Me: Problems in Canadian Medical Education,” shows a rare, deep, and penetrating acuity. He tells us that the idea that he was special was cultivated (and drilled into him - my words) by, no less, the faculty of his medical school. And this happens across the country. What were we thinking? The barely subtle message has some not so subtle negative consequences, as Sylvia Cruess and her team point out in a commentary on Liao’s letter, not the least of which is a loss of altruism, a core value of any profession, but especially medicine.

In “Physician Recruitment and Retention in New Brunswick: A Medical Student Perspective,” Mariah Giberson and her team explore physician recruitment and retention from the medical students’ viewpoints. The 158 medical students who completed the online survey indicated that job availability for both the medical student and the partner were the top factors when deciding where to practice. We wonder if that is a regional effect, and challenge other researchers to find out (then let us know).

Jerry Maniate and his team in “Supporting clinician educators to achieve ‘work-work balance’” highlighted the many tensions clinician educators face in balancing the sometimes competing demands of clinical, education, research, and administration tasks. You will have to read their paper to discover the “Four Ps” that support clinician educators’ performance and productivity through work-work balance.

Marlon Danilewitz et al. in “A Landscape Analysis of Leadership training in Postgraduate Medical Education (PGME) Training Programs at the University of Ottawa” showed that, while there is some agreement on the importance of leadership skills and training in postgraduate education, the “no time” excuse shows that there are far more pressing priorities. Who will identify the elephant in the room?

“Point-of-Care Ultrasound as a Competency for General Internists: A Survey of Internal Medicine Training Programs in Canada” by Jonathan Ailon et al. found that, while three quarters of internal medicine trainees and over half of General Internal Medicine faculty used point of care ultrasound clinically, the vast majority of residents and two thirds of faculty had received little or no training, an obvious discrepancy pointing to a clear need. How far behind practice will training lag?

In “Addressing Gaps in Physician Knowledge Regarding Transgender Health and Healthcare through Medical Education,” McPhail and colleagues wrote about the high rates of discrimination and related illnesses suffered by transgender persons (those persons whose sex at birth does not “match” their felt gender identity) due, in large part, to the denial of care by physicians. Interviewing both physicians and trans people, they found transphobia and a lack of physician knowledge, as reported both by trans people and by physicians, resulted in a denial of trans-specific care. They recommend trans health topics be included in medical education curricula.

Solmundson et al, in “Are we adequately preparing the next generation of physicians to prescribe exercise as prevention and treatment?” asked why few physicians provide exercise prescription in spite of the myriad benefits. Surveying UBC family medicine residents, they found (with a spectacular response rate of over 80% (319/396), that more than 95% felt prescribing physical activity would be important in their future practice while rating themselves “somewhat incompetent.” There appears to be a clear need here! Medical school curriculum committees and post-graduate program directors take note!

Wen Tay et al, in “Systems 1 and 2 thinking processes and cognitive reflection testing in medical students,” used the simple Cognitive Reflection Test (CRT) to measure the ability of 128 medical students to activate metacognitive processes and switch to System 2 (analytic) thinking where System 1 (intuitive) thinking might mislead them. Ten percent of students chose the intuitive, but incorrect answers to all three questions, suggesting those students generally relied on System 1 thinking. Approximately 44% of respondents answered all three questions correctly, indicating full analytical, System 2 thinking. While CRT performance may not predict their future expertise as clinicians, the test may be a useful educational exercise in helping students to understand (1) the importance of regulating their thinking when they do get out into clinical practice, and (2) that they should not believe everything they think.

Desrosiers and her co-authors have given us a timely study, “Curricular Initiatives that Enhance Student Knowledge and Perceptions of Sexual and Gender Minority Groups: a Critical Interpretive Synthesis.” With no accepted best practice for helping students learn to care for sexual and gender diverse groups, they set out to synthesize the relevant literature using a modified Critical Interpretive Synthesis. From thirty-one articles, they found that the multi-modal strategies that encouraged awareness of one’s lens and privilege in conjunction with facilitated communication seemed the most effective. They add that to move forward theoretically and practically we will need to draw on both the wider cultural competence literature and the sexual and gender diversity literature. Perhaps there is some *phronesis* for us all in their modest conclusion: helping our students with their formation, education and training may take a multi-model mentored approach to curriculum design and implementation.

“Learning-by-Concordance (LbC): Introducing undergraduate students to the complexity and uncertainty of clinical practice” by Fernandez and his team described the problem of the steep learning curve in early clerkship. The gap between preclerkship course content and the reality of clinical decision-making expected in clerkship can make your head spin. Deer caught in the headlights, anyone? The Learning-by-Concordance approach can bridge this gap by providing expert responses that students compare with their own, expert explanations and key-messages. The authors concluded that expert panel answers and explanations can contribute to the development of appropriate professional reasoning processes. But of course, the same could be said of many other practical application focused curricula. What’s stopping us from focusing more on clinical decision making? One barrier is the prevailing belief in medical education that students not only need, but must be buried by, truckloads of scientific facts before they can be trusted to learn even the basics of clinical decision making. There are many elephants in that room or perhaps just one very large immutable and immoveable mammoth!

